# The attribution of incentive salience to Pavlovian alcohol cues: a shift from goal-tracking to sign-tracking

**DOI:** 10.3389/fnbeh.2015.00054

**Published:** 2015-03-03

**Authors:** Chandra S. Srey, Jean-Marie N. Maddux, Nadia Chaudhri

**Affiliations:** Department of Psychology, Center for Studies in Behavioral Neurobiology/FRQS Groupe de Recherche en Neurobiologie Comportementale, Concordia UniversityMontreal, QC, Canada

**Keywords:** ethanol, autoshaping, motivation, conditioned stimulus, conditioned reinforcement, rat

## Abstract

Environmental stimuli that are reliably paired with alcohol may acquire incentive salience, a property that can operate in the use and abuse of alcohol. Here we investigated the incentive salience of Pavlovian alcohol cues using a preclinical animal model. Male, Long-Evans rats (Harlan) with unrestricted access to food and water were acclimated to drinking 15% ethanol (v/v) in their home-cages. Rats then received Pavlovian autoshaping training in which the 10 s presentation of a retractable lever served as the conditioned stimulus (CS) and 15% ethanol served as the unconditioned stimulus (US) (0.2 ml/CS; 12 CS presentations/session; 27 sessions). Next, in an operant test of conditioned reinforcement, nose pokes into an active aperture delivered presentations of the lever-CS, whereas nose pokes into an inactive aperture had no consequences. Across initial autoshaping sessions, goal-tracking behavior, as measured by entries into the fluid port where ethanol was delivered, developed rapidly. However, with extended training goal-tracking diminished, and sign-tracking responses, as measured by lever-CS activations, emerged. Control rats that received explicitly unpaired CS and US presentations did not show goal-tracking or sign-tracking responses. In the test for conditioned reinforcement, rats with CS-US pairings during autoshaping training made more active relative to inactive nose pokes, whereas rats in the unpaired control group did not. Moreover, active nose pokes were positively correlated with sign-tracking behavior during autoshaping. Extended training may produce a shift in the learned properties of Pavlovian alcohol cues, such that after initially predicting alcohol availability they acquire robust incentive salience.

## Introduction

Pavlovian cues that are associated with drugs of abuse can have robust and lasting influences on behavior. For example, Pavlovian drug cues attract attention (Hogarth et al., 2003; Field and Cox, 2008); evoke conditioned autonomic responses (Back et al., 2014); trigger drug craving (Litt and Cooney, 1999; Ramirez and Miranda, 2014); activate brain reward circuits (Childress et al., 1999); and influence relapse (Litt et al., 2000). Animal models of addiction and relapse confirm the vital role of Pavlovian drug cues in perpetuating drug use and abuse. In these models, environmental stimuli associated with drug availability can facilitate drug self-administration (Caggiula et al., 2001; Chaudhri et al., 2007) and prompt drug-seeking behavior after extinction (De Wit and Stewart, 1981) or abstinence (Grimm et al., 2001).

In addition to predicting the unconditioned stimulus (US), appetitive Pavlovian cues can serve as incentive stimuli (Robinson and Berridge, 1993), an acquired property that has been linked to their capacity to motivate drug use (Flagel et al., 2009). Interestingly, there appears to be considerable individual variation in the attribution of incentive salience to Pavlovian cues in rats (Robinson and Flagel, 2009). This conclusion is drawn from studies using a Pavlovian autoshaping procedure where a food pellet US is delivered immediately after presentation of a retractable lever, which serves as the conditioned stimulus (CS). Burgeoning research (Yager and Robinson, 2010, 2013; Flagel et al., 2011a; Meyer et al., 2012a; Anselme et al., 2013; Lesaint et al., 2014; Morrow et al., 2015) indicates that during presentation of the lever-CS a subset of rats approaches the location where the US is delivered, learned behavior referred to as “goal-tracking.” Another subset comes to approach and vigorously engage the lever-CS. This learned behavior, referred to as “sign-tracking,” is interpreted as evidence of the CS having acquired incentive salience. Importantly, although the lever-CS predicts the US in both goal-trackers and sign-trackers, only in sign-trackers does it gain incentive salience and become a “desired” stimulus (Robinson and Flagel, 2009). This inference is supported by the finding that the lever-CS serves as a conditioned reinforcer for a novel operant response only in rats that have been categorized as sign-trackers (Robinson and Flagel, 2009).

Using the Pavlovian autoshaping procedure it has been found that an individual's propensity to attribute incentive salience to a Pavlovian food cue correlates positively with susceptibility to the incentive motivational properties of Pavlovian drug cues. For example, rats that sign-track to a food cue are more likely to subsequently attribute incentive motivational properties to a cocaine cue (Meyer et al., 2012b), and are more sensitive to the influence of cocaine cues in cocaine self-administration and reinstatement tests (Yager and Robinson, 2013). Rats also sign-track to a CS that is associated with intravenous cocaine (Uslaner et al., 2006) or heroin infusions (Peters and De Vries, 2014), suggesting that Pavlovian drug cues acquire incentive motivational properties (Di Ciano and Everitt, 2004).

In people who drink alcohol, sensory stimuli like the smell and taste of alcohol can evoke conditioned responses, suggesting that such stimuli function as Pavlovian cues that predict alcohol. Preclinical research suggests that Pavlovian alcohol cues also acquire incentive salience; however, the use of food deprivation (Tomie et al., 2003) or sweetened alcohol (Krank, 2003) in these studies may have influenced the attribution of incentive salience to those cues. Food deprivation results in negative physiological energy balance, which can increase the incentive salience of caloric outcomes, such as ethanol (Fedorchak and Bolles, 1987; Lockie and Andrews, 2013). Furthermore, when presented with similar concentrations (5%) of ethanol and sucrose, rats lever press at higher levels to earn sucrose, suggesting greater willingness to work for the sweet solutions (Samson et al., 1982). Consequently, in the present study we used a Pavlovian autoshaping procedure with unsweetened, 15% ethanol as the US in rats that were not food or water deprived to test the hypothesis that Pavlovian alcohol cues acquire incentive salience. Goal- and sign-tracking responses were examined across 27 Pavlovian autoshaping sessions, after which the capacity of the lever-CS to reinforce a new operant response was examined across 4 tests for conditioned reinforcement.

## Materials and methods

### Subjects

Twenty-five male, Long-Evans rats (Harlan, Indianapolis, IN; 220–240 g on arrival) were used. Rats were single-housed in shoebox cages (44.5 × 25.8 × 21.7 cm) and given 1 week to acclimate to a controlled colony room environment (21.0°C; 44% humidity; 12-h light/dark cycle; lights on at 7:00 AM; all procedures conducted in the light phase). Each cage contained beta chip bedding (Sani Chips, Harlan) and a nylabone toy (Nylabones, Bio-Serv) for enrichment. Access to food (Agribrands, Charles River) and water was unrestricted throughout the experiment. All procedures were approved by the Concordia University Animal Research Ethics Committee and met the guidelines of the Canadian Council on Animal Care.

### Apparatus

Behavioral procedures were conducted in 12 conditioning chambers (ENV 009A; Med Associates Inc., St-Albans, VT) each enclosed within a ventilated, sound-attenuating cubicle. The door, back wall and ceiling of each chamber were made of clear polycarbonate, while the side walls, rod floor (ENV-009A-GF) and removable waste pan were made of stainless steel. A white house light (75W, 100 mA, ENV-215M) was centrally located on the upper left wall and a dual cup fluid port (ENV-200R3AM) was centrally located on the lower right wall. Ethanol was delivered into the port via polyethylene tubing using a 20 ml syringe mounted onto a syringe pump (PMH-100, 3.33 rpm) that was located outside the sound-attenuating cubicle. Disruption of an infrared beam across the opening of the port was used to measure entries into the port. For Pavlovian autoshaping training, a stainless steel retractable lever (4.8 × 1.9 cm; ENV-112M) was located 6.9 cm above the rod floor on either side of the port. The application of 25 grams of weight onto the lever produced recordable lever activation.

For the test of conditioned reinforcement the retractable levers were replaced with nose poke devices (ENV-114 BM) that were approximately 2.8 cm above the rod floor. Nose poke responses were measured by disruption of an infrared beam across the opening of the nose poke aperture. The fluid port was replaced with the left lever that had been used during Pavlovian autoshaping training. All other aspects of the conditioning chambers remained the same. The timing of all experimental events was controlled by a computer and Med PC-IV software (Med Associates, Inc.), which also recorded behavioral measures.

### Home-cage ethanol exposure

A 15% ethanol (v/v) solution was prepared using 95% ethanol and tap water. Rats were given access to 15% ethanol for 12 sessions using a 24 h, intermittent access, two-bottle choice procedure that induces high levels of ethanol consumption in outbred rats (Wise, 1973; Simms et al., 2008; Sparks et al., 2013). On Monday, Wednesday and Friday of each week, rats were weighed and then given access to 15% ethanol and water via two bottles on the home-cage. Ethanol was presented in a pre-weighed, 100 ml graduated cylinder and water was presented in a pre-weighed 400 ml plastic bottle. Both receptacles were sealed with identical rubber stoppers that contained metal sipper tubes that were inserted into the home-cage through the cage lid. Ball bearings within the sipper tubes were used to minimize spillage. At 24 h after placement on the cage lid, the ethanol cylinders and water bottles were removed and weighed, after which only the water bottles were placed back onto the cage lids. A total of 12 sessions in which rats had access to both ethanol and water simultaneously were conducted (3 per week).

To ensure that rats did not develop a side preference, the left or right placement of the ethanol cylinder and water bottle on the lid of the home-cages was alternated in each session. To account for spillage, ethanol cylinders and water bottles were placed onto two empty cages, and weighed at the same time as those on the experimental cages. Weight differences from the empty cages could be attributed to spillage, or evaporation. The average weight of water or ethanol from the empty cages in each session was subtracted from the corresponding data for each rat. The difference in bottle weights across the 24 h period was used to calculate ethanol intake (grams of ethanol consumed per kilogram of body weight) and ethanol preference (grams of ethanol solution consumed divided by grams of total fluid consumed in the same session).

Mean ethanol consumption for each rat was calculated across sessions 4 and 5. Starting on session 6, rats consuming less than 1.0 g/kg/24 h of ethanol were given a solution of 15% ethanol and 2% sucrose (15E2S) to briefly boost ethanol consumption. In total, one rat from the paired group and two rats from the unpaired group received 15E2S for two consecutive sessions, and one rat from the paired group received a single session of 15E2S.

### Behavioral procedures

#### Habituation

In order to reduce the effects of a novel environment on behavior, rats were habituated to the behavior room and conditioning chambers over 3 non-consecutive days. On day 1, rats were brought to the behavior room on a cart and left in their home-cages for 20 min. On day 2, rats were handled and weighed in the behavior room. On day 3, after being handled and weighed in the behavior room, rats were placed in the conditioning chambers for 20 min, during which time the house light was illuminated following a 1 min delay, and entries into the fluid port were counted.

#### Pavlovian autoshaping training

**Figure 1:**
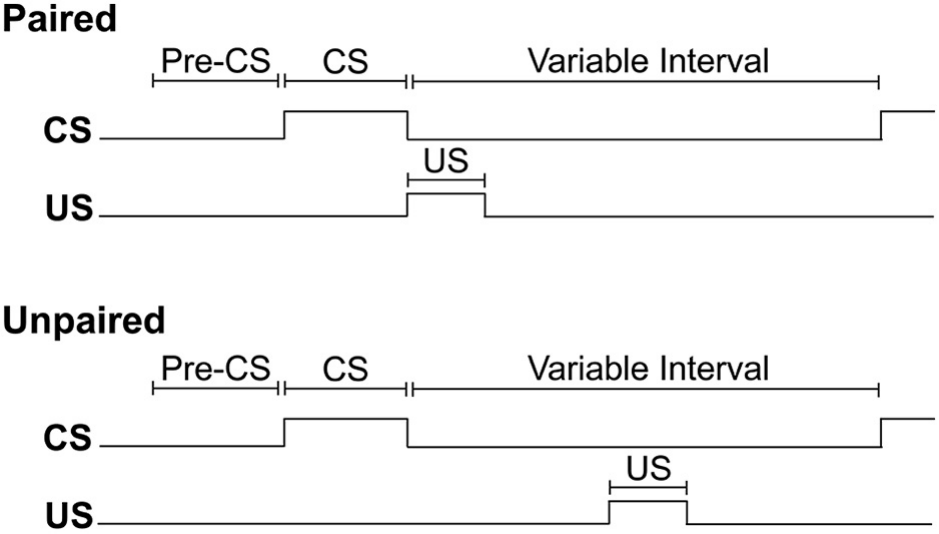
**The timing of experimental events during Pavlovian autoshaping sessions for paired and unpaired groups**. The conditioned stimulus (CS) occurred synchronously for both groups and consisted of the insertion of a lever into the conditioning chamber for 10 s. For the paired group, retraction of the lever-CS was immediately followed by the delivery of 0.2 ml of 15% ethanol unconditioned stimulus (US) across 6 s into a fluid port for oral consumption. For the unpaired group, US delivery occurred halfway between two lever-CS presentations. For both groups, the variable interval between offset of one lever-CS and onset of the next lever-CS was 260 s on average, excluding the 6 s over which ethanol was delivered.

For each training session, rats were weighed before being placed into the conditioning chamber. Initiation of the program resulted in a 2 min delay, followed by illumination of the house light to signal the start of the session. For all rats, insertion of a lever into the conditioning chamber for 10 s served as the CS. For rats assigned to the paired training group, the pump was activated for 6 s immediately upon retraction of the lever-CS. Pump activation delivered 0.2 ml of 15% ethanol into the fluid port for oral consumption. For rats assigned to the unpaired training group, ethanol delivery occurred mid-way between consecutive lever-CS presentations. In each session, both groups received 12 synchronized presentations of the lever-CS according to a 260 s variable time schedule. In this way, consecutive lever-CS presentations occurred at random with a variable inter-CS interval of 140, 260 or 380 s (the inter-CS interval does not include the 6 s period of pump activation). Thus, the paired group received CS-US pairings, while the unpaired group received equal exposure to the CS and US, but in an explicitly unpaired fashion. The timing of experimental events is depicted in Figure 1.

The total volume of ethanol delivered per session was 2.4 ml for each rat. Our method of ethanol delivery made it possible for ethanol that was not immediately consumed to accumulate in the port during a session. However, ports were checked after each session to verify that they were dry, and that the entire 2.4 ml of ethanol delivered per session had been consumed. Assignment to either paired or unpaired groups was counterbalanced based on ethanol g/kg, ethanol preference and body weight averaged across the last 2 sessions of home-cage ethanol exposure. Designation of the left or right lever as the conditioned stimulus during Pavlovian autoshaping training was counterbalanced across rats. Training sessions were conducted weekly on Monday, Wednesday and Friday, in order to mimic the timing of exposure to ethanol in the home-cage and to motivate ethanol consumption during Pavlovian autoshaping sessions. Each session lasted on average 61.2 min and 27 sessions were conducted.

#### Test of conditioned reinforcement

Approximately 48 h after the last Pavlovian autoshaping session, all rats underwent an operant test of conditioned reinforcement. Entries into one nose poke aperture (designated “active”) resulted in presentation of the lever-CS for 2.5 s, while entries into the other nose poke aperture (designated “inactive”) had no consequences. Assignment of either the left or right nose poke as the active aperture was counterbalanced according to lever assignment during Pavlovian training, as well as the average number of lever-CS activations, normalized port entries and total port entries made during the last two Pavlovian training sessions.

During tests for conditioned reinforcement, illumination of the house light occurred before rats were placed into the conditioning chamber. A test was initiated by the first active nose poke and lasted 30 min. In the event that a rat did not make an active nose poke, the test was terminated after 60 min. The first 3 active nose pokes were reinforced on a continuous reinforcement schedule (one lever-CS presentation per active nose poke). Subsequently, a variable ratio schedule of two (VR-2) took effect, whereby the lever-CS was presented after 1, 2, or 3 active nose pokes, according to a Latin square design (as in Olausson et al., 2004; Chaudhri et al., 2006; Lof et al., 2010). The use of a variable ratio schedule increases the unpredictability of CS presentations, which can help minimize within-session extinction.

A total of four consecutive tests of conditioned reinforcement were conducted in order to evaluate the longevity of this effect (Guy and Fletcher, 2013).

### Statistical analyses

#### Home-cage ethanol exposure

Dependent variables consisted of ethanol intake (g/kg/24 h; grams of ethanol consumed per kilogram of body weight) and ethanol preference (%; grams of ethanol solution consumed divided by grams of total fluid consumed in the same session), which were analyzed using repeated-measures analyses of variance (ANOVA) across *Session* (within-subject; 1-12) and *Group* (between-subject; paired or unpaired).

#### Pavlovian autoshaping training

To control for individual differences in baseline port entry behavior, a normalized port entry measure was calculated by subtracting port entries made during a 10 s pre-CS interval from port entries made during the corresponding lever-CS. The number of times that the lever-CS was activated during each presentation was recorded. In addition, latency to initially contact the lever-CS and latency to enter the fluid port upon presentation of the lever-CS were recorded. As each lever-CS presentation was 10 s long, rats that did not activate the lever-CS or make a port entry during a given lever-CS presentation were coded with 10 s latency. Therefore, dependent variables during Pavlovian autoshaping training consisted of the number of lever-CS activations (sign-tracking) and normalized port entries (goal-tracking), as well as latency to initially contact the lever-CS and latency to enter the fluid port upon presentation of the lever-CS. Data were analyzed using repeated-measures ANOVA across *Session* (1–27) and *Group* (paired or unpaired).

#### Test of conditioned reinforcement

The number of entries into active and inactive nose pokes, lever-CS presentations earned and lever-CS activations were recorded. Nose poke data were analyzed using repeated-measures ANOVA across *Test* (within-subject; 1–4), *Aperture* (within-subject; active or inactive) and *Group* (paired or unpaired). Lever-CS presentations and activations were analyzed across *Test* and *Group*.

Violations of homogeneity as indicated by Mauchly's test for sphericity were corrected for using Huynh-Feldt estimates, while violations of Levene's test for equality of variance were corrected for using an adjusted *p*-value. Significant interactions were pursued using independent samples *t*-tests to compare groups at given sessions, and paired samples *t*-tests to compare sessions within a group. All analyses used the statistical significance level of α = 0.05. Data were dropped for 1 rat that became aggressive and 1 rat that died before completing the study. Consequently, the final sample size consisted of 23 rats (*n* = 11 paired; *n* = 12 unpaired).

## Results

### Home-cage ethanol exposure

**Figure 2:**
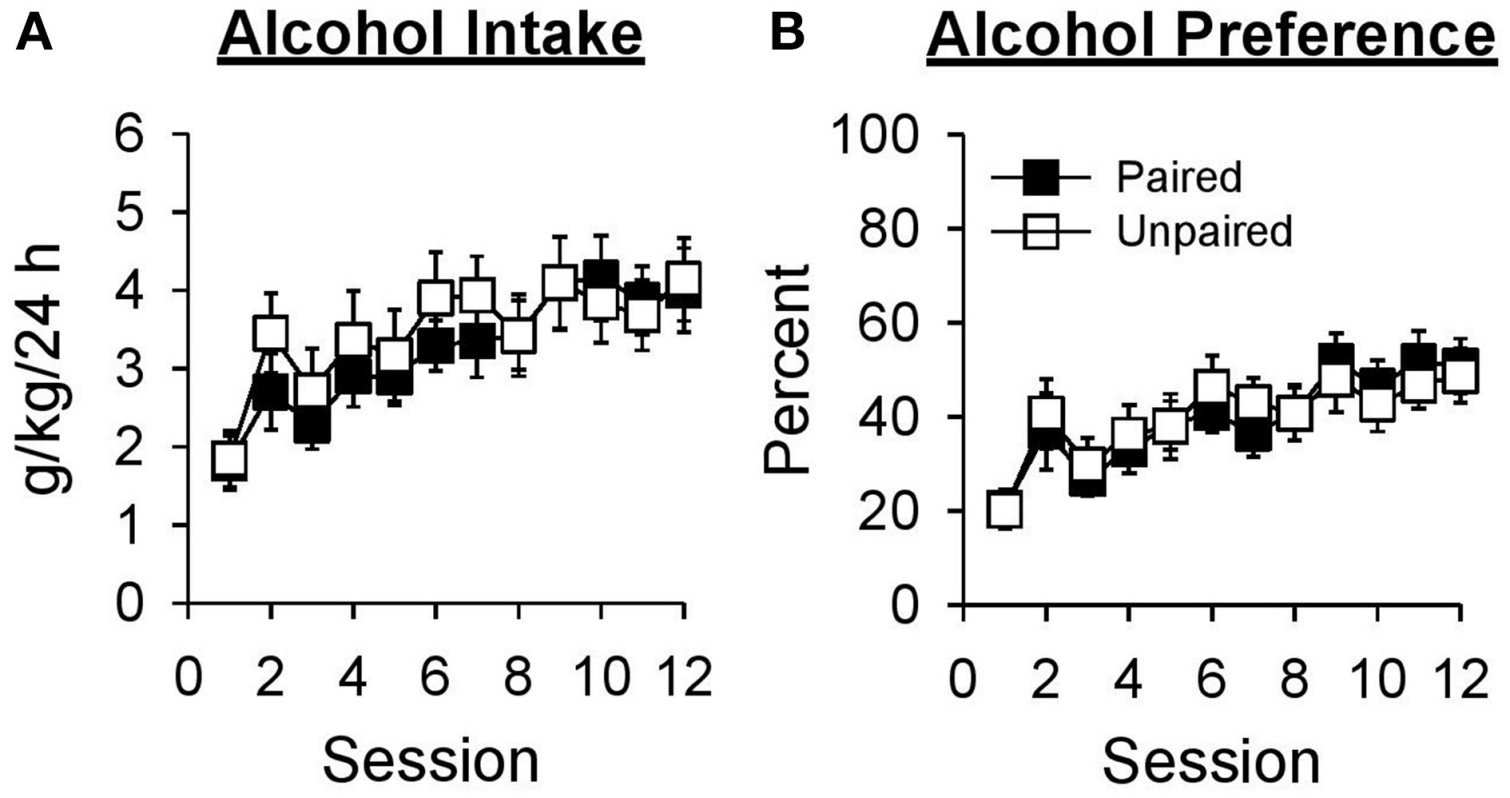
**Alcohol intake and preference increased across 12 sessions in which access to 15% ethanol was provided in the home-cage for 24 h**. In this and subsequent graphs, black symbols represent the paired group (*n* = 11) and white symbols represent the unpaired group (*n* = 12). Data are expressed as mean ± SEM for each session. **(A)** Alcohol intake in grams of ethanol consumed as a function of rat weight (g/kg/24 h). **(B)** Alcohol preference calculated as grams of ethanol solution consumed divided by grams of total fluid consumed in the same session and expressed as a percentage (%).

Alcohol intake increased across sessions in both groups [Figure 2A; Session, *F*_(11, 231)_ = 8.92, *p* < 0.001; Group, *F*_(1, 21)_ = 0.19, *p* = 0.670; Session × Group, *F*_(11, 231)_ = 0.54, *p* = 0.811]. Collapsed across group, alcohol intake was greater in session 12 than session 1 [*t*_(22)_ = −5.22, *p* < 0.001]. Alcohol preference (Figure 2B) also increased across sessions in both groups [Session, *F*_(11, 231)_ = 10.07, *p* < 0.001; Group, *F*_(1, 21)_ = 0.01, *p* = 0.932; Session × Group, *F*_(11, 231)_ = 0.45, *p* = 0.880]. Collapsed across group, alcohol preference was greater in session 12 than session 1 [*t*_(22)_ = −6.65, *p* < 0.001].

### Pavlovian autoshaping training

Alcohol intake (g/kg) in the first and last session of Pavlovian autoshaping training is presented in Table 1. The reduction in g/kg from session 1 to session 27 is attributable to the volume of alcohol delivered per session remaining constant, but rat weights increasing over the course of the experiment. There was no difference in g/kg as a function of group at either the start [session 1, *t*_(21)_ = −0.50, *p* = 0.619] or end [session 27, *t*_(21)_ = −1.12, *p* = 0.276] of Pavlovian autoshaping training.

**Table 1:** **Estimated alcohol intake (g/kg) in the first and last session of Pavlovian autoshaping training**.

	**Paired group (g/kg)**		**Unpaired group (g/kg)**	
	***M***	***SD***	***M***	***SD***
Session 1	0.67	0.01	0.68	0.02
Session 27	0.53	0.01	0.55	0.02

#### Port entries

**Figure 3:**
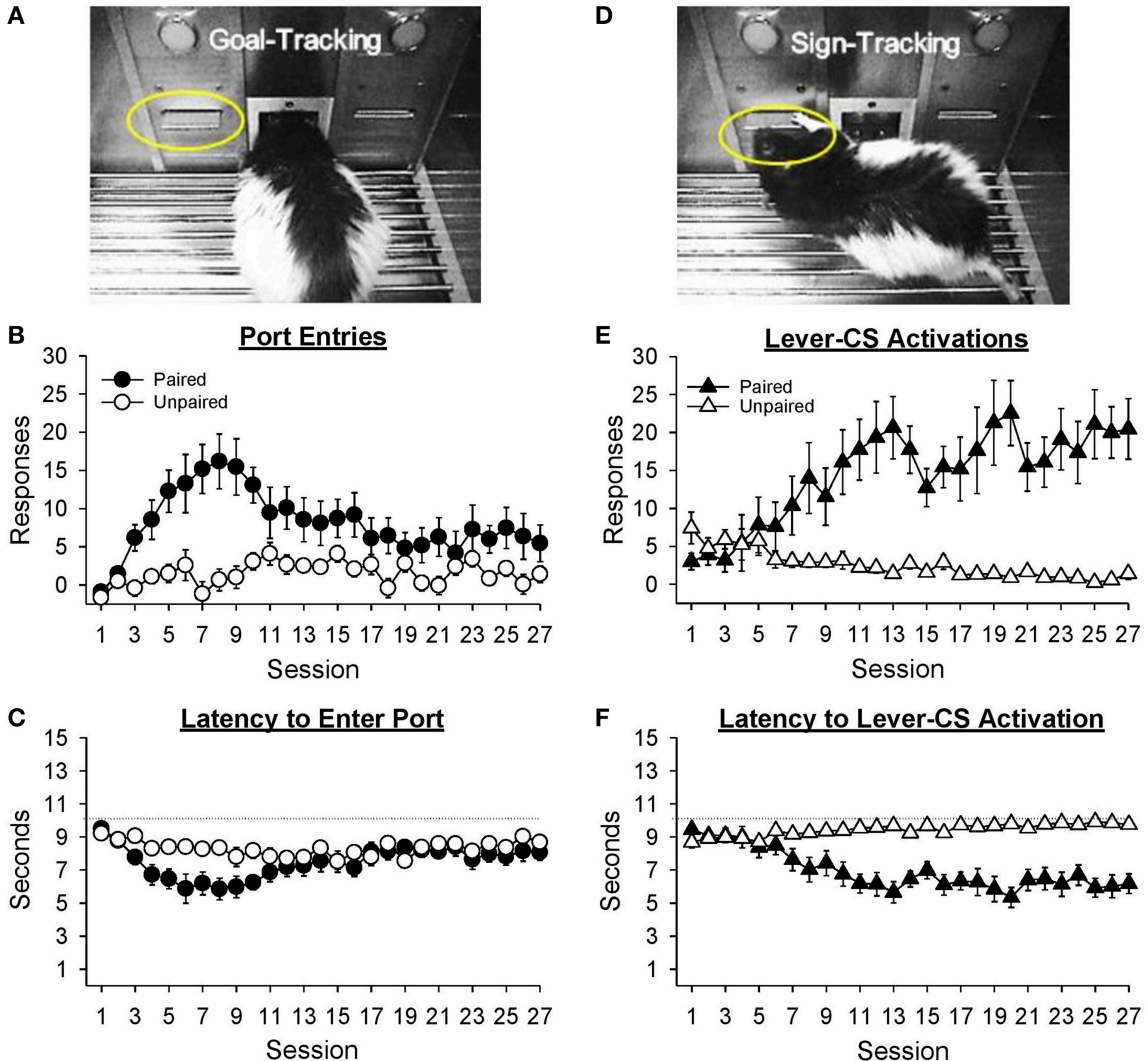
**Conditioned responding elicited by the lever-CS shifted from initial goal-tracking responses to robust sign-tracking behavior with extended Pavlovian autoshaping training**. Data are expressed as mean ± SEM for each training session. **(A)** Photograph depicting goal-tracking behavior, defined as entries into the fluid port during the lever-CS. **(B)** Normalized port entries during the lever-CS across session. To calculate a normalized measure that accounted for differences in baseline levels of behavior, port entries during a 10 s pre-CS interval were subtracted from port entries during the corresponding lever-CS. **(C)** Latency to enter the fluid port after presentation of the lever-CS. **(D)** Photograph depicting sign-tracking behavior, defined as activation of the lever-CS. **(E)** Number of lever-CS activations across session. **(F)** Latency to activate the lever-CS.

Figure 3A depicts a rat making a port entry (goal-tracking) response during presentation of the lever-CS. Normalized port entries during the lever-CS (Figure 3B) initially increased, then decreased as a function of session, only in the paired group [Session, *F*_(26, 546)_ = 4.85, *p* < 0.001; Group, *F*_(1, 21)_ = 11.44, *p* = 0.003; Session × Group, *F*_(26, 546)_ = 3.72, *p* < 0.001]. There were no between group differences in the number of normalized port entries made in session 1 [*t*_(21)_ = 0.68, *p* = 0.504] or session 27 [*t*_(21)_ = 1.57, *p* = 0.132]. Visual inspection of the data revealed session 8 to be the peak of this measure. On this session, the paired group made significantly more normalized port entries than the unpaired group [*t*_(21)_ = 4.02, *p* = 0.001]. In addition, for the paired group normalized port entries were similar in sessions 1 and 27 [*t*_(10)_ = −2.15, *p* = 0.057], but significantly higher in session 8 than session 1 [*t*_(10)_ = −4.47, *p* = 0.001]. For the unpaired group there were no differences between session 1 and either session 27 [*t*_(11)_ = −2.08, *p* = 0.061] or session 8 [*t*_(11)_ = −1.12, *p* = 0.286].

Latency to enter the port following presentation of the lever-CS (Figure 3C) initially decreased, and then increased across sessions only in the paired group [Session, *F*_(26, 546)_ = 5.14, *p* < 0.001; Group, *F*_(1, 21)_ = 4.31, *p* = 0.050; Session × Group, *F*_(26, 546)_ = 2.65, *p* = 0.001]. Latency to enter the port was similar across groups in sessions 1 [*t*_(21)_ = 1.03, *p* = 0.313] and 27 [*t*_(21)_ = −1.05, *p* = 0.307]. However, the paired group had faster latencies in session 27 than session 1 [*t*_(10)_ = 2.62, *p* = 0.026] and in session 8 than session 1 [*t*_(10)_ = 5.30, *p* < 0.001]. The unpaired group also had faster latencies in session 8 than session 1 [*t*_(11)_ = 2.73, *p* = 0.019], but similar latencies in sessions 1 and 27 [*t*_(11)_ = 1.41, *p* = 0.187].

#### Lever-CS activations

Sign-tracking to the lever-CS is shown in Figure 3D. Activations of the lever-CS (Figure 3E) increased across session in the paired group but decreased across session in the unpaired group [Session, *F*_(26, 546)_ = 2.51, *p* = 0.012; Group, *F*_(1, 21)_ = 21.42, *p* < 0.001; Session × Group, *F*_(26, 546)_ = 7.48, *p* < 0.001]. For the paired group, lever-CS activations were higher in session 27 compared to session 1 [*t*_(10)_ = −4.37, *p* = 0.001], whereas for the unpaired group lever-CS activations were lower in session 27 than session 1 [*t*_(11)_ = 3.04, *p* = 0.011]. Compared to the unpaired group, the paired group had more lever-CS activations in session 27 [*t*_(21)_ = 4.69, *p* = 0.001], but not in session 1 [*t*_(21)_ = −1.82, *p* = 0.083].

Latency to activate the lever-CS (Figure 3F) decreased as a function of session in the paired group [Session, *F*_(26, 546)_ = 3.98, *p* < 0.001; Group, *F*_(1, 21)_ = 32.48, *p* < 0.001; Session x Group, *F*_(26, 546)_ = 11.03, *p* < 0.001]. Compared to the unpaired group, the paired group had faster latencies in session 27 [*t*_(21)_ = −5.96, *p* < 0.001], but not in session 1 [*t*_(21)_ = 1.99, *p* = 0.060]. Moreover, the paired group had faster latencies in session 27 than session 1 [*t*_(10)_ = 5.31, *p* < 0.001], whereas the unpaired group was slower to activate the lever-CS in session 27 than session 1 [*t*_(11)_ = −3.58, *p* = 0.004].

**Figure 4:**
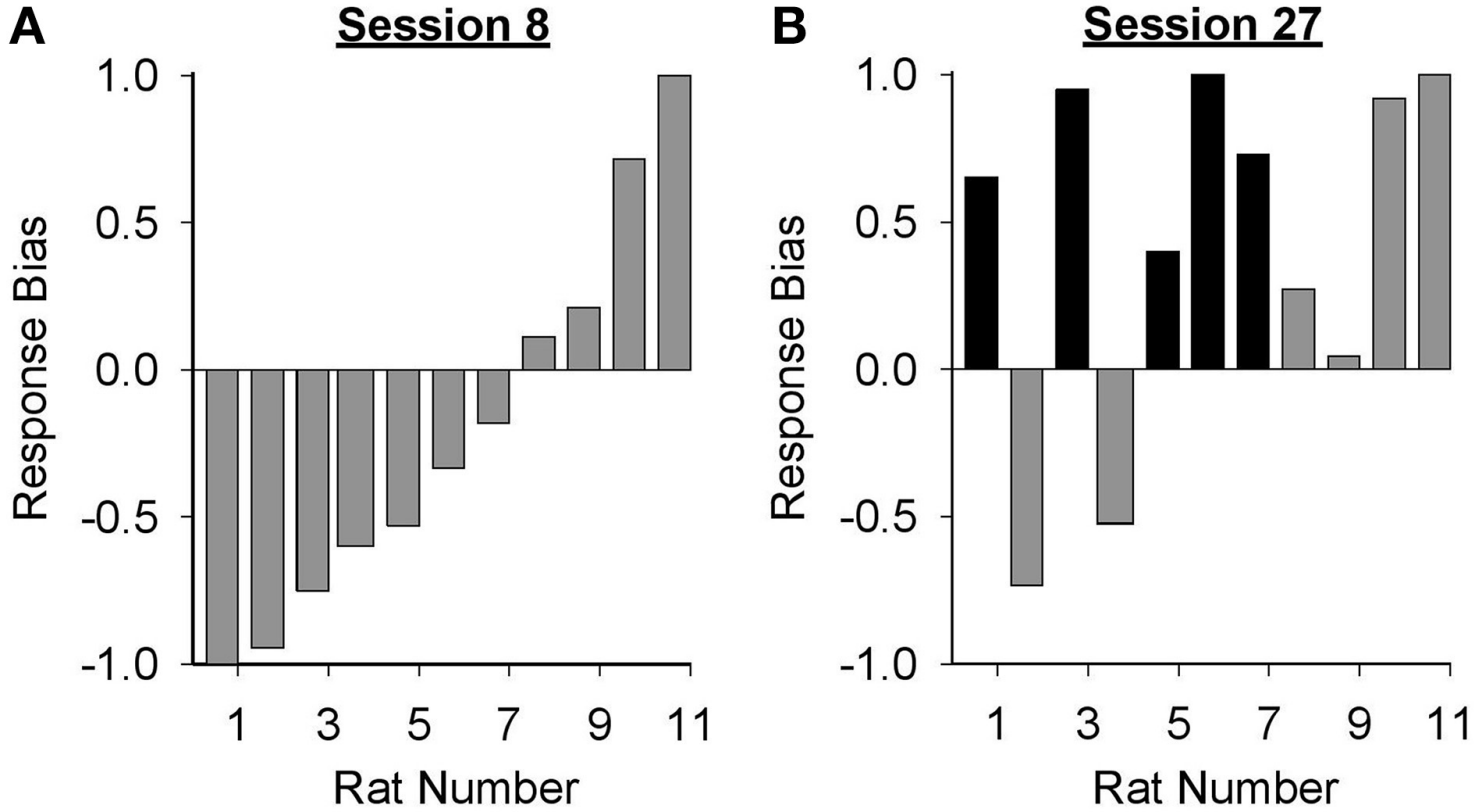
**Response bias shifted from primarily goal-tracking to predominantly sign-tracking with extended Pavlovian autoshaping training**. Response bias was calculated for each subject in the paired group using the formula: (number of lever-CS activations minus number of port entries)/(sum of lever-CS activations and port entries). The x-axis represents the identification number of individual rats represented in **(A)** session 8 and **(B)** session 27. A response bias score between −1 and 0 indicates a preference toward goal-tracking and a response bias score between 0 and 1 indicates a preference toward sign-tracking. Individual rats that demonstrated a shift in preference from goal-tracking in session 8 to sign-tracking in session 27 are depicted with black bars.

Data presented in Figure 3 suggest that the form of the conditioned response elicited by the lever-CS shifted from primarily goal-tracking to predominantly sign-tracking with extended Pavlovian autoshaping training. To visualize this shift, response bias (Meyer et al., 2012a) for individual rats in the paired group was calculated for sessions 8 (peak of goal-tracking) and 27 (last session of Pavlovian conditioning) using the following equation: (number of lever-CS activations minus number of port entries)/(number of lever-CS activations plus number of port entries). With this measure a proportion between −1 and 0 indicates more goal-tracking, and a proportion between 0 and 1 indicates more sign-tracking behavior. In session 8 (Figure 4A), 4 rats showed a bias for sign-tracking and 7 rats showed a bias for goal-tracking. By the last session (Figure 4B), 9 rats showed a bias for sign-tracking, which includes 5 rats that switched from a goal-tracking bias to a sign-tracking bias between sessions 8 and 27.

**Figure 5:**
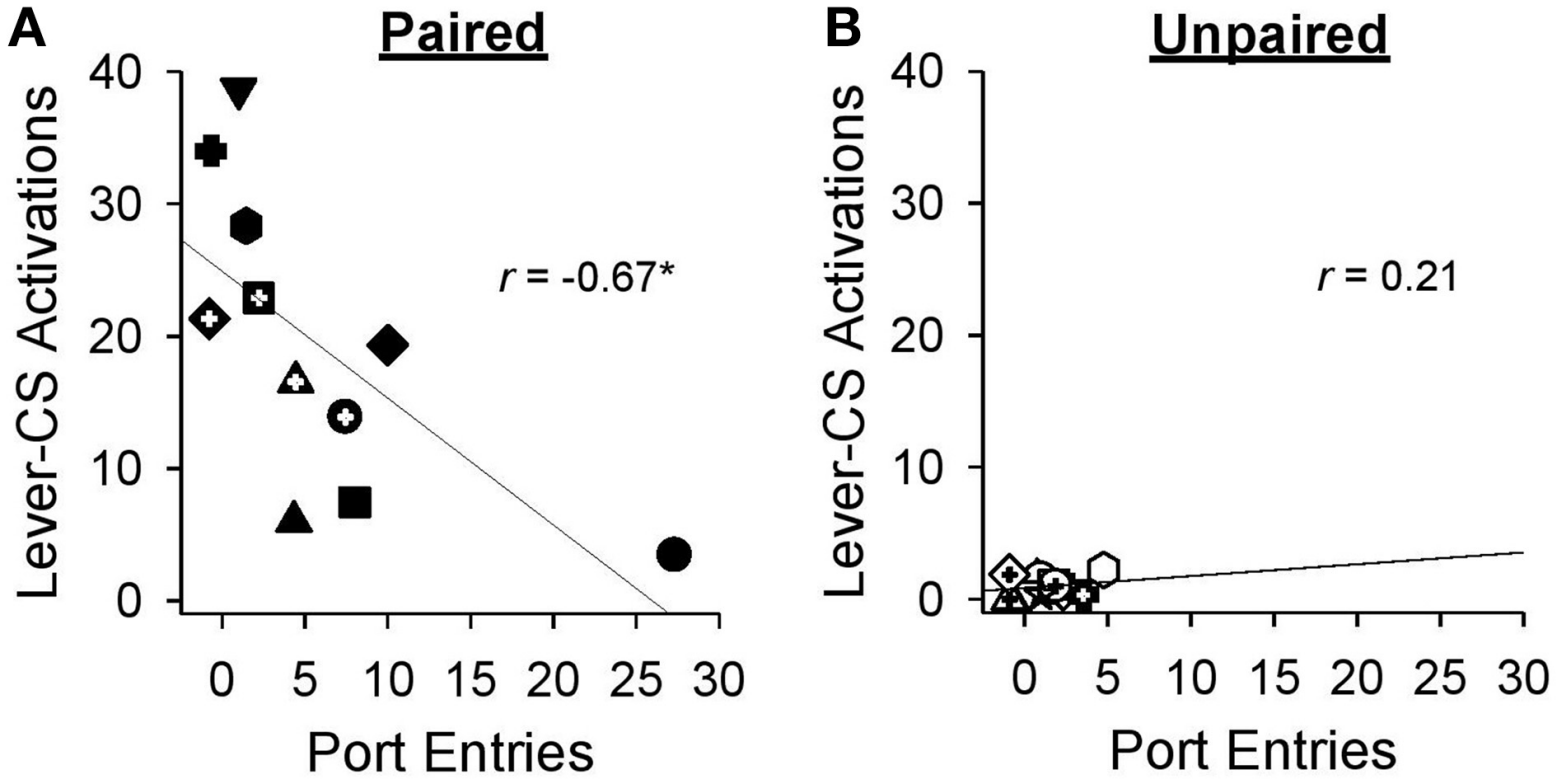
**Higher levels of sign-tracking behavior were associated with lower levels of goal-tracking responses at the end of Pavlovian autoshaping training**. Data represent mean ± SEM normalized port entries and lever-CS activations averaged across sessions 19–27. Each symbol represents data from an individual rat in **(A)** the paired group or **(B)** the unpaired group. Pearson's *r*-values are indicated in each graph. ^*^p < 0.05.

Pearson's correlations were used to probe the relation between sign-tracking and goal-tracking on behaviors averaged across the final sessions (19-27) of Pavlovian autoshaping training. By the end of training, greater lever-CS activations were associated with fewer normalized port entries [*r* = −0.67, *p* = 0.024] in the paired group (Figure 5A), but not in the unpaired group [Figure 5B; *r* = 0.21, *p* = 0.50].

#### Additional response measures

**Figure 6:**
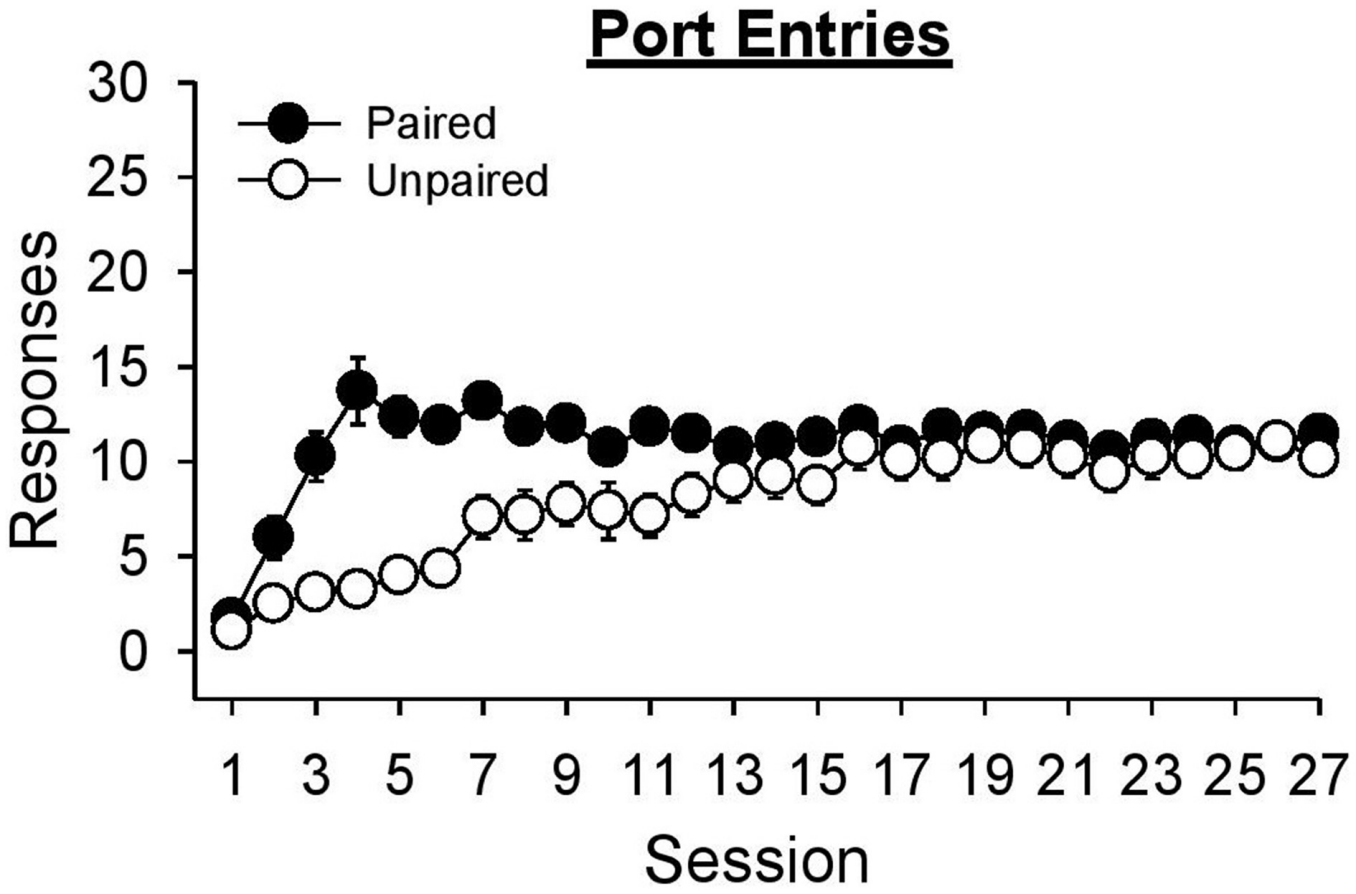
**Both paired and unpaired groups learned to approach the fluid port when alcohol was delivered during Pavlovian autoshaping training**. Data represent mean ± SEM number of port entries made during the 6 s when alcohol was delivered in to the fluid port.

Even though alcohol delivery in the unpaired group was not signaled by the lever-CS, rats in this group learned to enter the port when alcohol was delivered (Figure 6). An analysis of port entries made during the 6 s in which alcohol delivery occurred indicated that port entries increased across sessions [Session, *F*_(26, 546)_ = 20.37, *p* < 0.001] and were higher in the paired group than the unpaired group [Group, *F*_(1, 21)_ = 11.66, *p* = 0.003]. ANOVA also found a significant Session × Group interaction [*F*_(26, 546)_ = 8.06, *p* < 0.001]. Port entries were higher in session 27 than session 1 for both the paired [*t*_(10)_ = −13.86, *p* < 0.001] and unpaired group [*t*_(11)_ = −14.08, *p* < 0.001]. While there was no difference between groups at the start [session 1, *t*_(21)_ = 1.23, *p* = 0.233] or end [session 27, *t*_(21)_ = 1.44, *p* = 0.165] of training, the paired group made more port entries than the unpaired group in session 8 [t_(21)_ = 3.45, *p* = 0.005].

Additional analyses were conducted to determine if paired and unpaired groups differed with respect to the number of port entries made during two time intervals (10 and 60 s) that occurred after the presentation of the lever-CS (Supplementary Figure 1). Each time interval began 6 s after the lever-CS had been retracted, during which alcohol was delivered into the fluid port for the paired group. Port entries during both time intervals decreased across sessions, with no differences in the overall number of port entries made by either group.

Additional analyses were conducted to determine if the allocation of behavior as either goal-tracking responses or sign-tracking responses during the first half and last half of each lever-CS trial differed as a function of trial near the middle (session 7) or end (session 27) of Pavlovian autoshaping training (Supplementary Figure 2). No statistically supported patterns of behavior emerged from these analyses.

### Test of conditioned reinforcement

**Figure 7:**
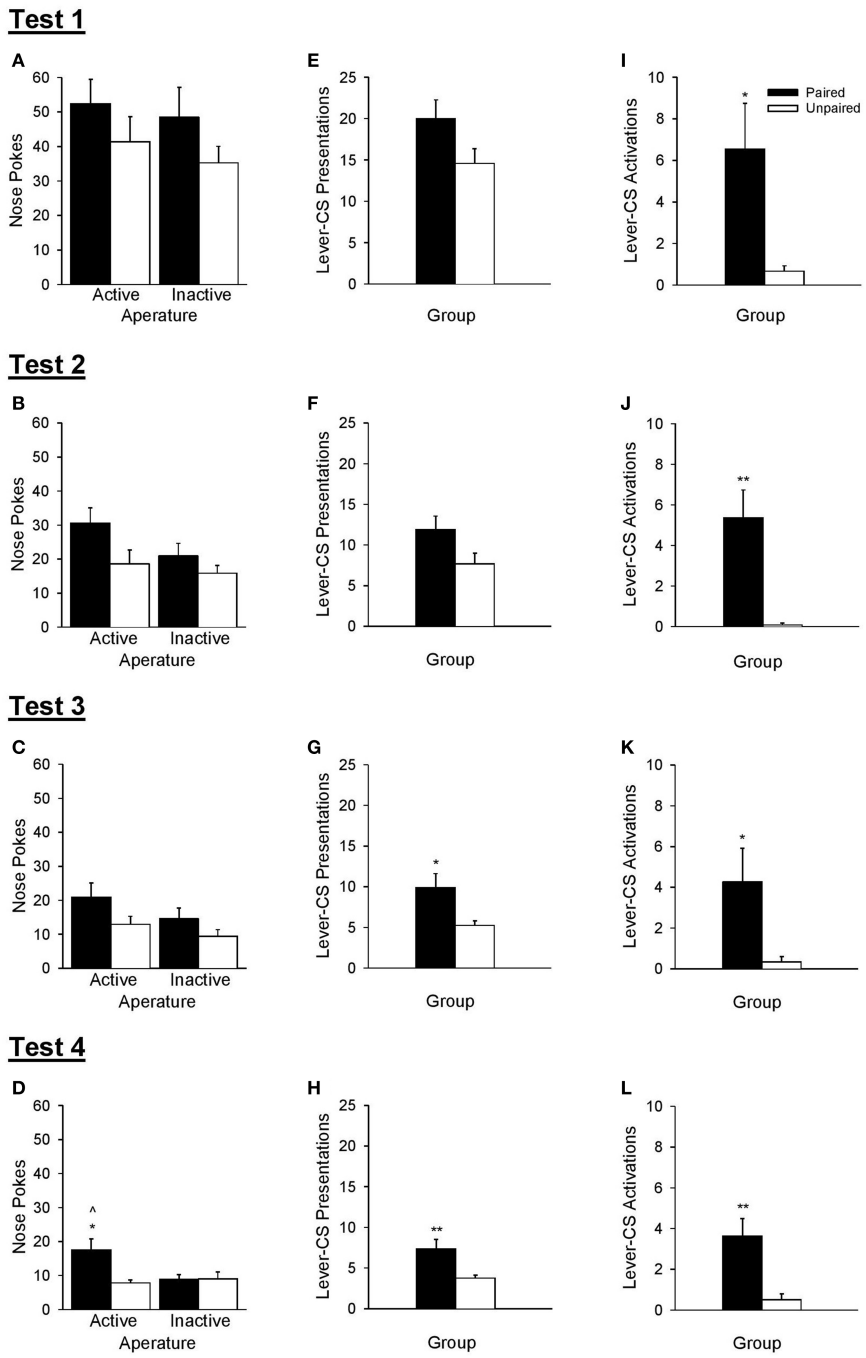
**A lever-CS that was previously paired with alcohol functioned as a conditioned reinforcer**. Black bars represent the paired group and white bars represent the unpaired group. Data are expressed as mean ± SEM for tests 1–4. **(A–D)** Number of nose pokes into the active and inactive apertures. **(E–H)** Number of lever-CS presentations earned. **(I–L)** Number of times the lever-CS was activated when it was presented as the result of active nose pokes. ^∧^*p* < 0.01, paired active vs. inactive. ^*^p < 0.05 and ^**^p < 0.01, paired vs. unpaired.

The ability of rats to discriminate between active and inactive nose poke apertures during operant tests of conditioned reinforcement (Figure 7) was used to verify that the lever-CS had acquired incentive value. The omnibus Test × Aperture × Group ANOVA revealed that the paired group made more nose pokes than the unpaired group [Group, *F*_(1, 21)_ = 5.91, *p* = 0.024] and that overall, nose poke responses decreased as a function of test [Test, *F*_(3, 63)_ = 58.35, *p* < 0.001]. No other significant main effects or interactions were found [all *F* < 2.86, *p* > 0.105].

As there was a main effect of test, each test of conditioned reinforcement was analyzed separately. These analyses revealed that there was no initial evidence of conditioned reinforcement in either group, but with repeated testing the lever-CS functioned as a conditioned reinforcer in the paired group in test 4. ANOVA conducted on nose poke responses for the first three tests (Figures 7A–C) indicated only a main effect of Group in test 3 [*F*_(1, 21)_ = 5.70, *p* = 0.027]. No other main effects or interactions were significant [all *F* < 4.26, *p* > 0.052]. However, ANOVA conducted on nose poke responses for test 4 (Figure 7D) indicated more responding in the active than the inactive aperture [Aperture, *F*_(1, 21)_ = 4.45, *p* = 0.047], more responding by the paired group than the unpaired group [Group, *F*_(1, 21)_ = 4.42, *p* = 0.048], as well as a significant interaction [Aperture × Group, *F*_(1, 21)_ = 8.56, *p* = 0.008]. The paired group discriminated between the active and inactive nose poke apertures in test 4 [*t*_(10)_ = 3.34, *p* = 0.008], but not in earlier tests [all *t* < 1.71, *p* > 0.119]. Conversely, the unpaired group did not discriminate between the active and inactive nose poke apertures in any of the 4 tests [all *t* < 1.10, *p* > 0.296]. Additionally, the paired group made more active nose pokes than the unpaired group only in test 4 [*t*_(21)_ = 3.01, *p* = 0.011], with no differences as a function of group in inactive nose pokes in any of the four tests [all *t* < 1.42, *p* > 0.172].

The number of lever-CS presentations earned did not differ across group in test 1 [Figure 7E, *t*_(21)_ = 1.90, *p* = 0.072], was marginally higher in the paired group in test 2 [Figure 7F, *t*_(21)_ = 2.04, *p* = 0.054] and significantly higher in the paired group than the unpaired group in test 3 [Figure 7G, *t*_(21)_ = 2.60, *p* = 0.023] and test 4 [Figure 7H, *t*_(21)_ = 3.03, *p* = 0.010].

Despite the lack of conditioned reinforcement in the first three tests, the paired group displayed greater lever-CS activations (Figures 7I–L) as early as test 1 [*t*_(21)_ = 2.66, *p* = 0.023], and this effect persisted in all subsequent tests [test 2, *t*_(21)_ = 3.85, *p* = 0.003; test 3, *t*_(21)_ = 2.36, *p* = 0.039; test 4, *t*_(21)_ = 3.40, *p* = 0.005].

**Figure 8:**
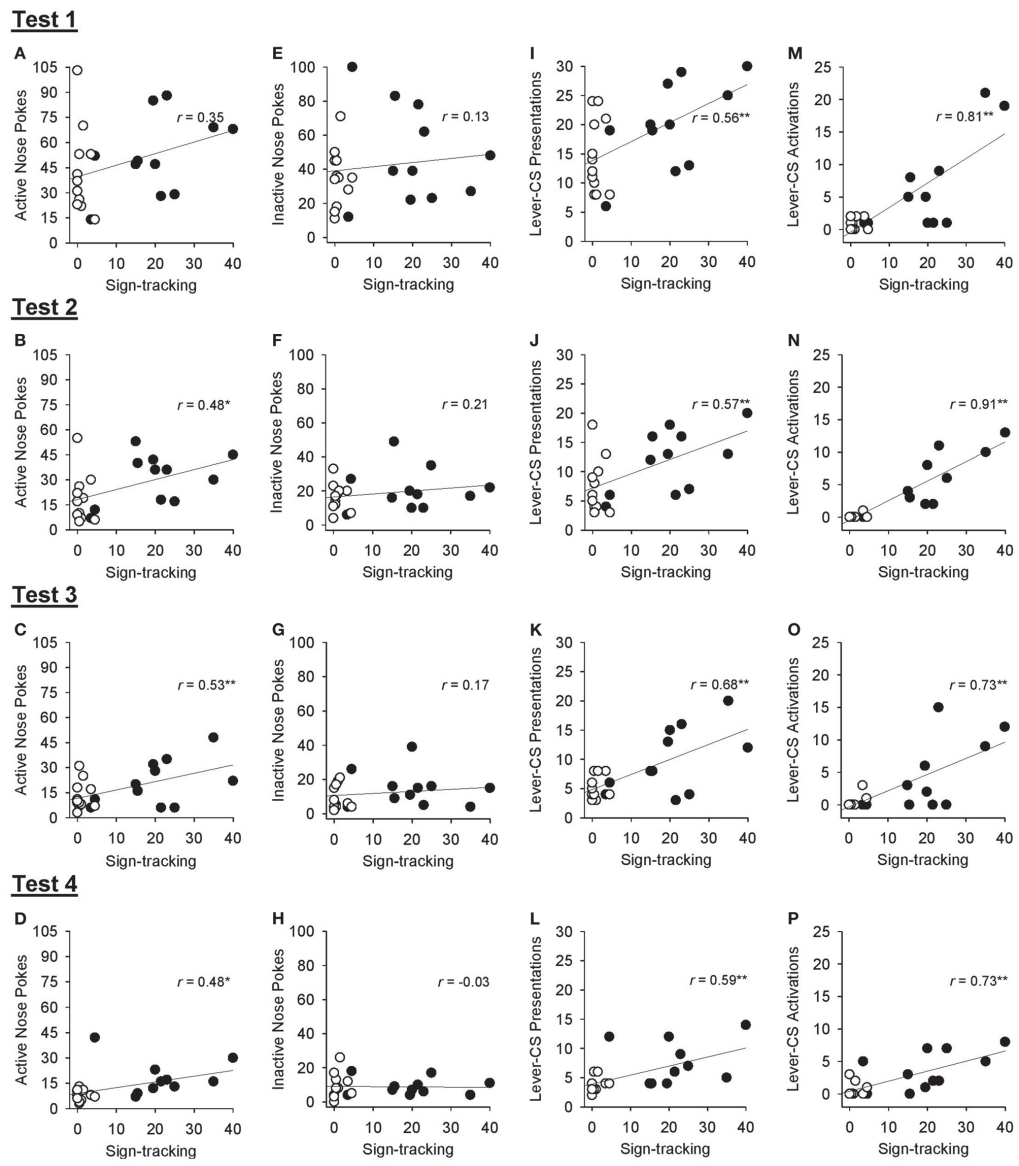
**Correlations between sign-tracking behavior at the end of Pavlovian autoshaping training and response measures obtained in each of the 4 tests for conditioned reinforcement in all rats**. In each graph, lever-CS activations averaged across sessions 26 and 27 of Pavlovian autoshaping training are plotted on the x-axis for each rat in the paired (black circles) and unpaired (white circles) groups. **(A–D)** Correlation between active nose poke responses and sign-tracking behavior. **(E–H)** Correlation between inactive nose poke responses and sign-tracking behavior. **(I–L)** Correlation between lever-CS presentations earned and sign-tracking behavior. **(M–P)** Correlation between lever-CS activations and sign-tracking behavior. ^*^p < 0.05 and ^**^p < 0.01.

Correlational analyses were conducted to assess the relation between sign-tracking averaged across sessions 26 and 27 of Pavlovian autoshaping training and various responses obtained during the tests of conditioned reinforcement in all rats. Excluding the first test of conditioned reinforcement, sign-tracking behavior was positively correlated with active nose pokes [Figures 8A–D; test 1, *r* = 0.35, *p* = 0.104; test 2, *r* = 0.48, *p* = 0.021; test 3, *r* = 0.53, *p* = 0.010; test 4, *r* = 0.48, *p* = 0.021] but not inactive nose pokes [Figures 8E–H; all *p* > 0.339]. Sign-tracking during Pavlovian autoshaping training was also positively correlated with the number of lever-CS presentations earned [Figures 8I–L; test 1: *r* = 0.56, *p* = 0.006; test 2, *r* = 0.57, *p* = 0.005; test 3, *r* = 0.68, *p* < 0.001; test 4, *r* = 0.59, *p* = 0.003] and number of lever-CS activations [Figures 8M–P; test 1, *r* = 0.81, *p* < 0.001; test 2, *r* = 0.91, *p* < 0.001; test 3, *r* = 0.73, *p* < 0.001; test 4, *r* = 0.73, *p* < 0.001] in each test of conditioned reinforcement.

## Discussion

We found that a Pavlovian cue associated with unsweetened alcohol acquired incentive salience, as measured by sign-tracking and conditioned reinforcement, in rats with unrestricted access to food and water. Presentations of a lever-CS that were paired with alcohol resulted in the rapid acquisition of goal-tracking behavior. Remarkably, with extended training goal-tracking diminished and robust sign-tracking behavior emerged. During tests of conditioned reinforcement, presentation of the lever-CS reinforced operant behavior only in rats that had previously received paired instances of the lever-CS and alcohol. These hitherto unreported findings suggest that extended training causes a shift in the acquired properties of Pavlovian alcohol cues, such that conditioned stimuli that predict alcohol eventually become transformed into powerful incentive stimuli.

This interpretation is supported by converging evidence across multiple variables. From sessions 1–8, goal-tracking behavior indexed by normalized port entries during the lever-CS increased rapidly in the paired but not unpaired group, suggesting that the lever-CS became a conditioned stimulus that predicted alcohol delivery for the paired group. In parallel, latency to enter the fluid port during lever-CS presentations decreased in the paired group. From sessions 1–8, sign-tracking behavior as indexed by lever-CS activations also increased in the paired group and latency to activate the lever-CS decreased. Interestingly, with continued training goal-tracking responses triggered by the lever-CS decreased across sessions 9–27, until this measure no longer differed between groups. Over the same time course, sign-tracking responses in the paired group continued to increase and eventually stabilize, with a corresponding decrease in latency to lever-CS activation. Response bias scores for the paired group support a shift from primarily goal-tracking in session 8 to predominantly sign-tracking in session 27. Moreover, correlational analyses indicate that in session 27 rats with a greater propensity for sign-tracking were less likely to make goal-tracking responses. These novel findings suggest that before a Pavlovian alcohol cue can be attributed with incentive salience, it first has to serve as a reliable predictor of alcohol availability. Alternately, the development of predictive and incentive properties of Pavlovian alcohol cues may occur on different time courses that are not interdependent.

Data from the tests for conditioned reinforcement verified that the lever-CS became attributed with incentive salience. The paired group made more active nose pokes than inactive nose pokes in test 4, whereas this measure did not differ for the unpaired group. This pattern of behavior indicates that the lever-CS was not intrinsically reinforcing, because it only supported discriminated nose poke responding in rats for which the lever-CS had previously been paired with alcohol. The observation that conditioned reinforcement was not statistically significant until test 4 is likely because the novelty of the nose poke apertures stimulated high levels of indiscriminate responding that masked the conditioned reinforcement effect. More recently, we have observed conditioned reinforcement at test 1 in rats that were briefly habituated to both nose poke apertures 24 h before the test (Maddux and Chaudhri, unpublished data). Providing limited access to the novel operant response devices before tests of conditioned reinforcement has been used to reduce the influence of exploratory behavior on test data (Fletcher et al., 2002; Meyer et al., 2014).

Importantly, when compared to the unpaired group, the paired group activated the lever-CS more frequently when it was presented as a result of active nose pokes on all four conditioned reinforcement tests. Also, sign-tracking behavior at the end of Pavlovian autoshaping training was positively correlated with the number of active nose pokes in all but test 1, and with number of lever-CS presentations earned and frequency of lever-CS activations in all four tests. These data indicate for the first time that a Pavlovian cue associated with unsweetened, voluntarily consumed alcohol can function as a conditioned reinforcer in the absence of food or water deprivation. They support the hypothesis that Pavlovian cues associated with alcohol can become imbued with incentive salience, making them motivationally attractive stimuli (Bindra, 1974; Robinson and Berridge, 1993). In addition, they corroborate evidence indicating that rats identified as sign-trackers typically exhibit conditioned reinforcement, whereas rats identified as goal-trackers do not (Robinson and Flagel, 2009).

The question of whether or not Pavlovian alcohol cues acquire incentive salience as measured through sign-tracking behavior and conditioned reinforcement has been examined previously. In one of the first efforts to address this question, rats that received paired presentations of an auditory CS with intragastric infusions of ethanol subsequently pressed a lever to earn CS presentations (Smith et al., 1977). However, the absence of an inactive lever or an unpaired control group renders these data inconclusive. In another study (Krank et al., 2008), rats that were initially exposed to an ethanol/saccharin solution subsequently underwent Pavlovian conditioning sessions in which a light-CS was paired with unsweetened ethanol. Results obtained across 8 sessions indicated higher overall levels of approach and contact to the light-CS in paired vs. unpaired groups. Although approach and contact both decreased as a function of session, these measures along with entries into the fluid magazine where alcohol was delivered remained significantly elevated in the paired group, compared to the unpaired group, in session 8. With extended training it is conceivable that sign-tracking responses directed toward the light-CS may have continued to increase, with a corresponding reduction in goal-tracking behavior.

Using lever-CS activation as the only index of sign-tracking behavior in the present study may not have captured the full range of possible sign-tracking behaviors, which can include a variety of responses such as approach without physical interaction with the CS (Difeliceantonio and Berridge, 2012). As we did not quantify approach responses directed toward the lever-CS, the acquisition of sign-tracking behavior could have been underestimated. Similarly, rats may have interacted with the lever-CS, but without applying enough force on the lever to activate it. Replications of this assay would benefit from video recordings to better characterize the nature of sign-tracking behavior. The role of adventitious instrumental contingencies in the development of sign-tracking behavior also warrants further research. To address whether sign-tracking behavior is under primarily Pavlovian or instrumental control, an omission procedure can be used, in which lever activation cancels the delivery of the US. Sensitivity to an omission procedure is interpreted as evidence that an instrumental contingency contributes to behavior, whereas relative insensitivity is interpreted as evidence that the conditioned response is the result of a Pavlovian association. Sign-tracking studies that have utilized omission schedules with natural unconditioned stimuli support the prevailing view that sign-tracking behavior is primarily governed by Pavlovian learning (Williams and Williams, 1969; Stiers and Silberberg, 1974; Atnip, 1977; O'Connell, 1979; but see Sanabria et al., 2006). Future studies should include an omission procedure to determine if instrumental contingencies contribute to sign-tracking behavior using the current paradigm. Finally, to assess whether the observed shift from goal-tracking to sign-tracking behavior is specific to an alcohol cue, a comparison of the emergence of goal-tracking and sign-tracking to a cue for natural unconditioned stimuli should be considered. Our preliminary data using 10% sucrose as the unconditioned stimulus in the current paradigm suggest that this shift is not exclusive to an alcohol cue (Vo et al., unpublished data).

In the present study, rats drank high levels of alcohol in the home-cage before the start of behavioral training. Alcohol intake on the last session of home-cage exposure averaged 4.07 ± 0.37 g/kg/24 h, which is comparable to published reports (Simms et al., 2008; Sparks et al., 2013) and interpreted as robust alcohol consumption in outbred rats. Using similar procedures, we showed previously that rats consume enough alcohol within the first 30 min of access in the home-cage to produce measureable blood alcohol levels that correlate positively with oral intake (Chaudhri et al., 2008). Thus, it is likely that rats experienced the pharmacological effects of alcohol during the home-cage exposure phase. During each hour-long Pavlovian autoshaping session, 2.4 ml of 15% ethanol was distributed across 12 US presentations of 0.2 ml each. Collapsed across group, estimates of oral alcohol intake ranged from 0.68 ± 0.01 (mean ± SEM) in session 1 to 0.54 ± 0.01 g/kg in session 27, with the across session decrease in g/kg attributable to rats gaining weight over the course of the experiment. Alcohol intake within this range of values produces measurable levels of blood alcohol in operant self-administration procedures (Carrillo et al., 2008). However, because we did not assay blood alcohol levels it remains to be determined if rats experienced the pharmacological effects of alcohol during Pavlovian autoshaping training. Alternately, goal-tracking and sign-tracking behavior could have been acquired through second-order conditioning, with the smell and taste of each alcohol presentation serving as a first-order conditioned stimulus. This interesting possibility warrants further investigation.

Dopamine signaling is important for the attribution of incentive salience to appetitive Pavlovian cues (Robinson and Berridge, 1993; Berridge and Robinson, 1998). Published studies indicate that in rats identified as sign-trackers, striatal dopamine release elicited by a lever-CS increases across sessions, with a diminishing dopamine response to the food-pellet US (Flagel et al., 2011b). Conversely, the lever-CS and US continue to trigger small increases in dopamine across sessions in goal-trackers (Flagel et al., 2011b). In addition, blocking dopamine receptors in the nucleus accumbens core markedly reduces the expression of sign-tracking, but not goal-tracking behavior (Saunders and Robinson, 2012). Thus, sensitization of the dopamine system may be one mechanism underlying the gradual transformation of Pavlovian alcohol cues into incentive motivational stimuli.

Pavlovian cues that predict alcohol likely motivate alcohol consumption during the transition from casual drinking to heavy drinking and alcohol abuse. Sensory stimuli associated with alcohol evoke conditioned responses (Sinha and O'Malley, 1999; Field and Duka, 2002), indicating an acquired capacity to predict alcohol. If these sensory stimuli also acquire incentive salience then drinking behaviors that eventually lead to alcohol intoxication may be maintained by the conditioned reinforcing properties of such cues. Our data suggest that sign-tracking behavior directed toward visual cues associated with alcohol (e.g., glassware containing alcohol) might help to initiate and maintain alcohol consumption. In support of this hypothesis, the type of glassware in which alcohol is served can influence the rate of drinking (Attwood et al., 2012), and in a rodent model of drinking behavior pairing a sipper tube that contains alcohol with a food US can evoke sign-tracking responses directed toward the sipper that promote alcohol consumption (Tomie et al., 2002).

In conclusion, we report that a Pavlovian alcohol cue can become transformed from a conditioned stimulus that predicts alcohol availability to one that is imbued with strong incentive motivational properties. The gradual emergence of sign-tracking behavior suggests that the cue may first need to predict alcohol, before it acquires incentive salience and becomes desirable. Additional research aimed at understanding the mechanisms that mediate the transition from goal-tracking responses to sign-tracking behavior is needed. Such studies may be useful in advancing our capacity to prevent cues that predict alcohol from acquiring incentive salience and facilitating alcohol-seeking behavior and relapse.

### Conflict of interest statement

The authors declare that the research was conducted in the absence of any commercial or financial relationships that could be construed as a potential conflict of interest.
