# Supplementary material for: The attribution of incentive salience to Pavlovian alcohol cues: a shift from goal-tracking to sign-tracking
Source: Front Behav Neurosci. 2015 Mar 3;9:54. doi: 10.3389/fnbeh.2015.00054 (PMC4347508; doi:10.3389/fnbeh.2015.00054)
Supplement: Supplementary file 1 [file Presentation1.PDF]

Supplementary Material

The attribution of incentive salience to Pavlovian alcohol cues: a shift from goal-tracking to sign-tracking

C. S. Srey[1], J.M. Maddux[1], and N. Chaudhri[1]*

[1]Center for Studies in Behavioral Neurobiology/FRQS Groupe de recherche en neurobiologie comportementale, Concordia University, Department of Psychology, Montreal, QC, Canada

*** Correspondence:** Corresponding Author, CSBN/GRNC, Concordia University, Department of Psychology, 7141 Sherbrooke Street West, Montreal, QC, H4B-1R6, Canada.
nadia.chaudhri@concordia.ca

1. Supplementary Figures

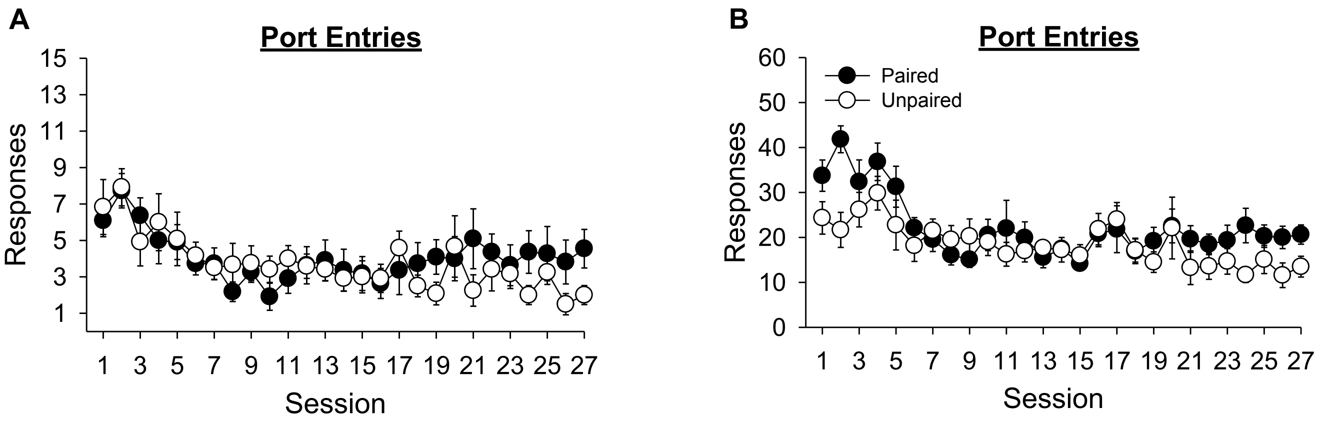

1.1. Figure Legend

Supplementary Figure 1 Port entries made during two time intervals that were not signaled by the lever-CS. Each interval began 6 seconds after the lever-CS had been retracted, during which ethanol was delivered into the fluid port for the paired group. **(A)** Mean ± SEM number of port entries during a 10 second interval. Port entries decreased across session, with no differences between groups [Session, $F_{(26, 546)} = 4.14$, $p < 0.001$; Group, $F_{(1, 21)} = 0.18$, $p = 0.674$; Session x Group: $F_{(26, 546)} = 1.14$, $p = 0.317$]. **(B)** Mean ± SEM number of port entries during a 60 second interval. Port entries decreased across sessions [Session, $F_{(26, 546)} = 6.58$, $p < 0.001$]. There was no difference in the overall number of port entries made by paired and unpaired groups [Group, $F_{(1, 21)} = 1.94$, $p = 0.178$], but there was a significant Session x Group interaction [$F_{(26, 546)} = 1.83$, $p = 0.025$]. Follow-up *t*-tests indicated a significant reduction in port entries in session 27 compared to session 1 for both paired [$t_{(10)} = 3.75$, $p = 0.004$] and unpaired [$t_{(11)} = 3.14$, $p = 0.009$] groups. Independent samples *t*-tests indicated that the paired group made more port entries than the unpaired group in 4 out of the 27 Pavlovian autoshaping training sessions [session 2, $t_{(21)} = 4.05$, $p = 0.001$; session 24, $t_{(21)} = 2.70$, $p = 0.013$; session 26, $t_{(21)} = 2.26$, $p = 0.035$; session 27, $t_{(21)} = 2.25$, $p = 0.035$].

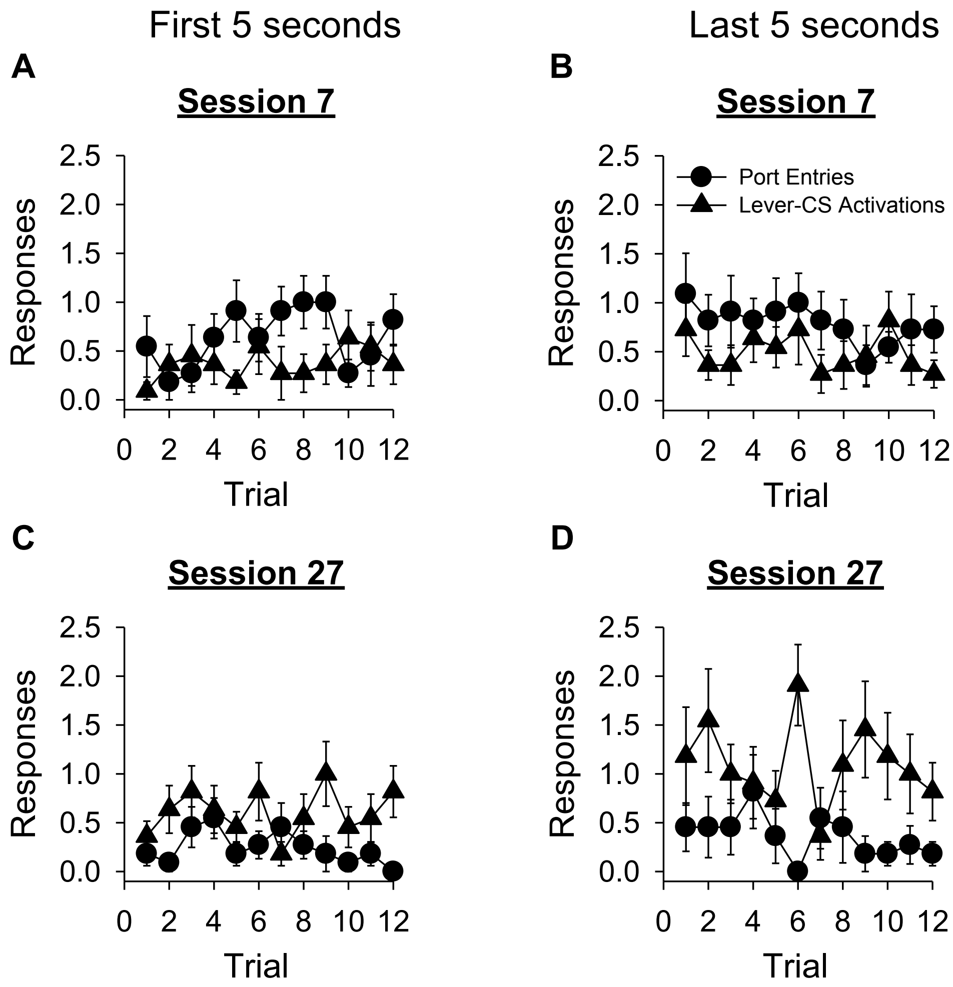

1.2. Figure Legend

Supplementary Figure 2 The allocation of behavior as either goal-tracking responses or sign-tracking responses during the first half and last half of each lever-CS trial did not differ as a function of trial during Pavlovian autoshaping training. Data represent the mean ± SEM number of port entries (circles) and lever-CS activations (triangles) made during the first 5 seconds and last 5 seconds of each lever-CS trial in the paired group. Session 7 represents one of the earliest sessions in which sign-tracking behavior started to emerge and session 27 represents a session in which the distribution of responses between sign-tracking and goal-tracking behavior is established. Separate ANOVA were conducted within each session and time block to analyze the distribution of each response type (port entries vs. lever-CS activations) as a function of CS trial. In each analysis, there was no main effect of Trial [all $F_{(11,110)} < 1.15$, all $p > 0.332$], no main effect of Response Type [all $F_{(1,10)} < 4.12$, all $p > 0.070$] and no Trial x Response Type interaction [all $F_{(11,110)} < 1.79$, all $p > 0.065$]. **(A)** First 5 seconds of each lever-CS trial in session 7. **(B)** Last 5 seconds of each lever-CS trial in session 7. **(C)** First 5 seconds of each lever-CS trial in session 27. **(D)** Last 5 seconds of each lever-CS trial in session 27.
